# One-step generation of error-prone PCR libraries using Gateway^® ^technology

**DOI:** 10.1186/1475-2859-11-14

**Published:** 2012-01-30

**Authors:** Antoine Gruet, Sonia Longhi, Christophe Bignon

**Affiliations:** 1 Architecture et Fonction des Macromolécules Biologiques (AFMB), UMR 7257 CNRS and Aix-Marseille University, 163, Avenue de Luminy, Case 932, 13288 Marseille, Cedex 09, France

**Keywords:** Cloning, Sub-cloning, Gateway^®^, Directed evolution, GFP, Error-prone PCR, epPCR, Library, Screening

## Abstract

**Background:**

Error-prone PCR (epPCR) libraries are one of the tools used in directed evolution. The Gateway^® ^technology allows constructing epPCR libraries virtually devoid of any background (*i.e*., of insert-free plasmid), but requires two steps: the BP and the LR reactions and the associated *E. coli *cell transformations and plasmid purifications.

**Results:**

We describe a method for making epPCR libraries in Gateway^® ^plasmids using an LR reaction without intermediate BP reaction. We also describe a BP-free and LR-free sub-cloning method for in-frame transferring the coding sequence of selected clones from the plasmid used to screen the library to another one devoid of tag used for screening (such as the green fluorescent protein). We report preliminary results of a directed evolution program using this method.

**Conclusions:**

The one-step method enables producing epPCR libraries of as high complexity and quality as does the regular, two-step, protocol for half the amount of work. In addition, it contributes to preserve the original complexity of the epPCR product.

## Background

Gateway^® ^is an appealing technology because its cloning efficiency is close to 100% [1]. This feature is particularly welcome when dealing with numerous target genes, for instance in Structural Genomics. Unfortunately, high throughput gene expression following gene cloning in Structural Genomics programs has also revealed that many recombinant proteins are insoluble in *E. coli *thereby precluding their crystallization and their study by X-ray crystallography. Among the different techniques used to overcome this insolubility problem, one is directed evolution. The use of directed evolution for improving recombinant protein solubility can be summarised as follows. A random library of mutants generated by error-prone PCR (epPCR) and/or DNA shuffling [2] is screened for variant proteins more soluble than the wild-type (*wt*) protein. To that end, the mutated DNA sequences may be expressed as fusion proteins with a C-terminal "solubility reporter" such as the green fluorescent protein (GFP) [3]. To assess the solubility gain provided by the mutations, the mutated coding sequences are then sub-cloned from the solubility reporter expression plasmid to a GFP-free expression plasmid and the solubility of the tag-free variant is compared to that of the tag-free *wt *protein expressed under the same conditions.

Although the Gateway^® ^technology is less used in directed evolution than in Structural Genomics programs, it has been nevertheless successfully applied in a directed evolution study that made use of both epPCR and DNA shuffling [4]. The evolved Tobacco Etch Virus (TEV) protease exhibited significantly higher solubility than the *wt*TEV protease. Incidentally, this study also revealed a few weak points that seemed to be specifically associated with the use of the Gateway^® ^technology rather than with the screening process or the protein to evolve. In particular, *(i) *the number of expression clones was found to be relatively small, as also reported in another study [5]; *(ii) *the generation of epPCR and DNA shuffling libraries was labor intensive because of the need for BP and LR reactions to be carried out, and of the corollary transformations and intermediate plasmid medium preparations [6]; *(iii) *the subcloning of the coding sequence of selected mutants from the reporter expression plasmid to a non-reporter expression plasmid was also time-consuming because of the same requirements.

While the first of the drawbacks listed above can be easily addressed by transforming expression cells by electroporation, addressing the other two requires devising a novel cloning and sub-cloning strategy. With the specific purpose of overcoming these limitations while maintaining the obvious advantages of the Gateway^® ^technology, we devised a method that allows eliminating the BP step from the generation of the library and both the BP and LR steps from the sub-cloning process. We applied this method to generate a diversity library of the intrinsically disordered C-terminal domain of the measles virus nucleoprotein (N_TAIL_) [7,8] as a first step towards the dissection of the molecular mechanisms underlying its interaction with the C-terminal X domain (XD, aa 459-507) of the viral phosphoprotein [9-17]. A split-GFP reassembly assay [18-20] was used to screen the library and to identify clones with novel binding properties.

## Results

### 1) Generation of an epPCR library

**Figure 1:**
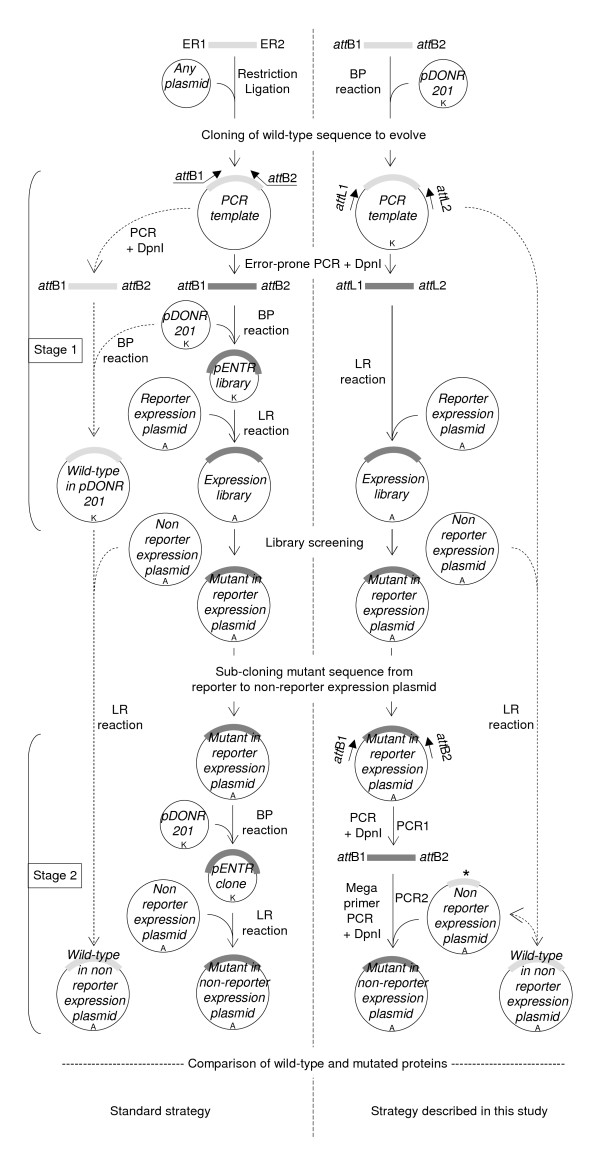
**Overview of the method**. The left flowchart is the standard strategy and the right flowchart is the strategy described in this study. Brackets on the left indicate the two stages of the strategy: the epPCR library construction (stage1) and the sub-cloning of mutant inserts from reporter to non-reporter expression plasmid (stage 2). ER1 and ER2 denote restriction sites used to clone the sequence to evolve and create the PCR template of the standard strategy. Inner arrows with continuous lines are the core of the method. Outer arrows with dashed lines indicate the pathways used to transfer the *wt *sequence to the non-reporter expression plasmid, and (in the right flowchart only) to create the internally deleted *wt*N_TAIL _in pDEST17O/I. Mutated and *wt *sequences are represented by thick dark and light grey lines, respectively. The internally deleted *wt *sequence is denoted by an asterisk. Antibiotic resistance markers are indicated: A, ampicillin resistance; K, kanamycin resistance.

The conventional procedure for generating epPCR libraries using the Gateway^® ^technology comprises two recombination reactions (BP and LR) [4]. We first addressed the question as to whether each recombination reaction and associated *E. coli *cell transformation decreased the complexity of a given library. A typical Gateway^® ^recombination reaction can be described as the transfer of an insert from a donor to a non-recombined acceptor to yield a recombined acceptor. Therefore, the library complexity loss can be evaluated by comparing the number of colonies provided by: *(i)* a theoretical experiment made of a 100% efficient LR reaction (*i.e*. a reaction where all the non-recombined acceptor (*i.e*., Gateway^® ^plasmid before LR reaction) molecules are used to yield recombined acceptors (*i.e*., Gateway^® ^plasmids after LR reaction)) followed by a 100% efficient cell transformation (*i.e*. a transformation where all recombined acceptor molecules are uptaken by cells and where each cell uptakes one recombined acceptor molecule); *(ii*) an actual cell transformation by a recombined acceptor; *(iii*) an actual cell transformation by an actual LR reaction using the same donor construct (*i.e*., the other substrate of the LR reaction) and the same non-recombined acceptor as in the previous two instances. The results of this comparison are reported in Table 1. Since 25 fmoles of acceptor correspond to 1.55 × 10^10 ^molecules, if the LR reaction and cell transformation were each 100% efficient, then one should obtain 1.55 × 10^10 ^colonies per 25 fmoles of input acceptor. However, transforming *E. coli *cells by electroporation with 25 fmoles of recombined acceptor provided a mean value of 18.7 × 10^7 ^colonies. Assuming that each cell uptakes only one plasmid molecule, this means that *E. coli *cells electroporation was responsible for a ~80-fold drop of the theoretical (maximal) number of clones in this experiment. In addition, when *E. coli *cells were electroporated with an LR reaction mixture using the same amount of the same acceptor, the average number of colonies was 2.7 × 10^7 ^(Table 1). This means that the recombination reaction was by itself responsible for an additional ~7 fold efficiency drop. Incidentally, this latter comparison provided a direct measure of the efficiency of the LR reaction, which could be determined to be approximately 14% under the conditions used in this experiment. In conclusion, each step made of a recombination reaction followed by transformation of *E. coli *by electroporation reduced the number of clones with respect to what could be expected from a 100% efficient LR reaction combined with a 100% efficient cell transformation. Thus, skipping one recombination reaction and the associated *E. coli *transformation in the Gateway^® ^cloning process would be expected to better preserve the original library complexity than the classical two step approach (BP followed by LR), particularly when heat-shock is used instead of electroporation to transform *E. coli *cells (Table 1). Incidentally, this would also reduce the risk of biasing the pENTR library (*i.e*., the BP reaction product, see stage 1 in the left panel of Figure 1) by unbalanced clone growing during the culture in the presence of kanamycin. On the basis of this observation, we devised a new "single recombination" method for constructing epPCR libraries.

**Table 1:** Assessment of the efficiency of *E.coli *transformation by different DNA species

Experiment	Transforming DNA	Number of clones
Theoretical	LR reaction	1.55 × 10^10^

Electroporation	Recombined acceptor^1^	18.7 (± 7.2) × 10^7^
	LR reaction^2^	2.7 (± 1.5) × 10^7^

Heat shock	Recombined acceptor^1^	10.8 (± 2.1) × 10^5^
	LR reaction^2^	1.8 (± 0.4) × 10^5^

The number of colonies obtained after *E. coli *transformation by a recombined acceptor^1^, or by an LR reaction^2 ^is reported. T7pRos *E. coli *cells were either electroporated as described in "Methods", or transformed by heat-shock. Transformed cells were selected on ACplates. The results are the mean value and standard deviation of three independent transformations using the same LR reaction

^1^25 fmoles of pNGG-N_TAIL _(Table 2). ^2^using 25 fmoles of non recombined acceptor plasmid (pNGG, Table 1) and 100 fmoles of linear N_TAIL _coding sequence flanked by *att*L recombination sites (Figure 1, stage 1, right panel)

**Table 2:** Plasmid constructs used in this study

Name	Insert	Source	Purpose
pET11a-link-NGFP	None	[7]	pNGG backbone
pNGG	Gateway cassette	This study	Library screening
pNGG-N_TAIL_	N_TAIL_	This study	Positive control
pNGG-Stop-N_TAIL_	Stop-N_TAIL_	This study	Negative control
pDONR-Stop-N_TAIL_	Stop-N_TAIL_	This study	LR substrate
pDONR-N_TAIL_	N_TAIL_	This study	epPCR substrate
pET11a-Z-NGFP	Leucine zipper	[7]	Positive control
pMRBAD-Z-CGFP	Leucine zipper	[7]	Positive control
pMRBAD-link-CGFP	None	[7]	CGFP cloning backbone
pMRBAD-XD-CGFP	XD	This study	N_TAIL _interacting partner
pDEST17O/I-N_TAIL_	N_TAIL_	This study	idN_TAIL _PCR template
pDEST17O/I-idN_TAIL_	Internally deleted N_TAIL_	This study	PCR2 template

The strategy we have devised is described in Figure 1 (right flowchart) and compared to the regular strategy (left flowchart and reference [4]). It includes not only the generation of the library (Figure 1, stage 1), but also the sub-cloning of selected mutant sequences from the expression plasmid used for screening the random mutant library (referred to as "reporter expression plasmid" throughout the text) to a different expression plasmid (referred to as "non-reporter expression plasmid") (Figure 1, stage 2) allowing the variant proteins of interest to be expressed without the screening tag (such as GFP). In the regular strategy [4], generating and then cloning an epPCR library into a Gateway^® ^reporter expression plasmid requires three steps (Figure 1, stage 1, left flowchart). In the first step, the epPCR product (dark grey in Figure 1, stage 1) is generated using two primers hybridizing to the 5' and 3' ends of the coding sequence desired to be subjected to epPCR and flanked at their respective 5' end by Gateway^® ^*att*B1 and *att*B2 recombination sites (25-mer), and a PCR template consisting of the *wt *target sequence (light-grey in Figure 1, stage 1). In the second step, the epPCR product is inserted in a shuttle plasmid (pDONR201) by mean of a BP reaction. After *E. coli *transformation, an aliquot is spread on kanamycin plates to assess the library complexity, and the remaining is grown in liquid culture (100 ml). A plasmid medium preparation is then performed to recover the cloned library (pENTR library) from the liquid culture. In the third step, an aliquot of the plasmid medium preparation is used in an LR reaction to transfer the inserts from the entry clones of the pENTR library to a reporter expression plasmid. The LR reaction is performed as described above for the BP reaction except that ampicillin is used instead of kanamycin. In this procedure, obtaining a high-complexity library relies on the efficiency of two critical steps, namely the BP and LR reactions. In the strategy we developed, the epPCR library is generated using the *att*L1 (25-mer) and *att*L2 (24-mer) primers and the *wt *coding sequence already cloned in the pDONR201 plasmid as PCR template. Primers *att*L1 and *att*L2 respectively hybridize to the "Forward- and Reverse-priming sites" located upstream and downstream the *att*L1 (100 bp) and *att*L2 (100 bp) Gateway^® ^recombination sites (Additional file 1: Figure S1). As a result, the epPCR product is flanked by full-length *att*L1 and *att*L2 recombination sites and hence can be directly used in the LR reaction. This procedure enables to eliminate three steps of the regular approach: *(i) *the BP reaction that transfers the epPCR product to pDONR to create the pENTR library; *(ii) *the transformation of *E. coli *with the pENTR library; *(iii) *the purification (plasmid medium preparation) of the latter. Beyond the advantage related to the reduction of the number of steps, this procedure also offers the advantage of preserving the library complexity as shown above.

### 2) Sub-cloning of selected mutated sequences

**Figure 2:**
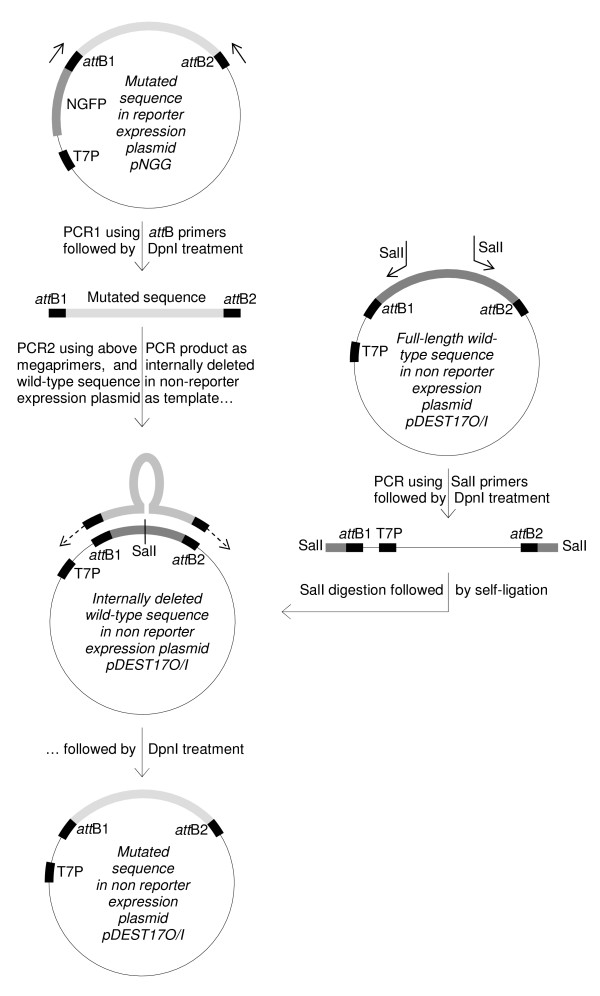
**Schematic of the sub-cloning of a mutated sequence from reporter to non-reporter expression plasmid**. From top to bottom, the DNA fragments amplified by PCR between the T7prom and *att*B2 primers are 1,046 bp, 333 bp, and 560 bp in length, respectively. On the right is illustrated how inverse PCR was used to internally delete 227 bp from the *wt*N_TAIL _sequence in pDEST17O/I. Plasmids are not at scale.

After mutants of interest have been selected, their coding sequence must generally be sub-cloned from the reporter expression plasmid used to screen the epPCR library to another non-reporter expression plasmid (Figure 1, stage 2). In the regular strategy [4], this procedure essentially follows the same process as that used to create the random library (compare stage 1 and stage 2 of Figure 1, left flowchart). In the first step, the mutated coding sequence is transferred from the reporter expression plasmid to pDONR201 by mean of a BP reaction. *E. coli *cells are transformed with the BP reaction mixture, and transformed cells are selected on kanamycin plates. Given the efficiency of Gateway^® ^transfer [1], few colonies need to be analyzed. The plasmid of each colony is purified by mini-preparation, and then checked for the presence of the proper insert generally by PCR using the *att*L1 and *att*L2 primers. The plasmid of one positive pENTR clone is then used as substrate in an LR reaction to transfer the coding sequence from pDONR to the non-reporter expression plasmid. The regular strategy works well when few mutants have to be processed, but we realized that it was impractical in directed evolution projects where many mutated coding sequences have to be sub-cloned in parallel from reporter to non-reporter expression plasmid(s). Therefore, we devised the alternative strategy depicted in the right flowchart of Figure 1, stage 2 and in more details in Figure 2. This strategy could be described as a hybrid method between MEGAWHOP [21] and RF cloning [22]. MEGAWHOP was devised to clone a random library of linear DNA. In practice, a library of mutated linear DNA is used as a complementary pair of megaprimers in a PCR experiment. The PCR template is the non mutated DNA sequence borne by the expression plasmid to be used for screening the library. Since the hybridization mismatch between the *wt *sequence borne by the expression plasmid and each mutated sequence to sub-clone (*i.e*., the mutation rate) is low compared to the homology, the megaprimers hybridize very efficiently to the *wt *sequence. During the PCR elongation steps, the whole plasmid is copied. After amplification, the PCR template is degraded by DpnI treatment. The final product is the random library of DNA borne by the expression plasmid. RF cloning also aims at inserting a linear double strand DNA into a plasmid, but uses a different approach. In contrast to MEGAWHOP, not the whole length of the linear DNA to clone is used for hybridization, but only 24 base pairs at both ends which are designed to be complementary to the cloning site of the plasmid. When this linear DNA is used in a PCR experiment with the cloning plasmid as template, each 24 base flanking extension hybridizes to its complementary sequence on the plasmid, resulting in a linear amplification of the plasmid during the PCR elongation step. After amplification the PCR template is degraded by DpnI treatment. The final product is the linear DNA borne by the plasmid. Our sub-cloning technique relies on the use of megaprimers, as in MEGAWHOP, and on the annealing of only the ends of the megaprimers, as in RF cloning. The method consists of two steps. In the first step, the mutated coding sequence is PCR amplified using the *att*B1 and *att*B2 primers and the reporter expression plasmid bearing the mutated coding sequence as template (PCR1, Figures 1 and 2). After DpnI treatment to remove methylated (*i.e*. parental) DNA, the PCR product flanked by full-length *att*B1 and *att*B2 Gateway^® ^recombination sites is used as a pair of complementary megaprimers in a second PCR step that uses an internally deleted form of the *wt *coding sequence borne by the non-reporter expression plasmid as template (PCR2, Figures 1 and 2). The reason for using an internally deleted sequence as PCR2 template is explained in the last paragraph of the results section. The result of this second PCR is the full-length mutated coding sequence in the non-reporter expression plasmid.

### 3) Application of the method to the N_TAIL_-XD interaction project

We have applied the above-described strategy to few directed evolution projects (B. Coutard, F. Vincent, unpublished data), including the one reported herein. In view of gaining insights into the N_TAIL_-XD interaction, we generated a mutant library of N_TAIL _and used a split GFP re-assembly assay to screen variants with altered interaction abilities [18-20]. In this method, the sequence coding for one of the two interacting partners under study is inserted in the prokaryotic expression vector pET11a-link-NGFP so as to lead to its expression as a fusion protein with the N-terminal half of GFP (NGFP). The coding sequence of the second interacting partner is inserted in another prokaryotic expression vector (pMRBAD-link-CGFP) thus leading to its expression as a fusion protein with the C-terminal half of GFP (CGFP). Both fusion proteins are then co-expressed in *E. coli*. If the two proteins of interest interact with each other in the bacterium, their interaction allows the two GFP halves to re-associate and reconstitute the functional (*i.e*., fluorescent) GFP. Thus, fluorescent bacteria denote an interaction between the two partners, with the fluorescence intensity being proportional to the affinity of this interaction. In practice, a library of N_TAIL _random mutants was inserted in a modified pET11a-link-NGFP vector (see below), and mutants were screened for altered interacting abilities with *wt*XD expressed from pMRBAD-XD-CGFP.

**Figure 3:**
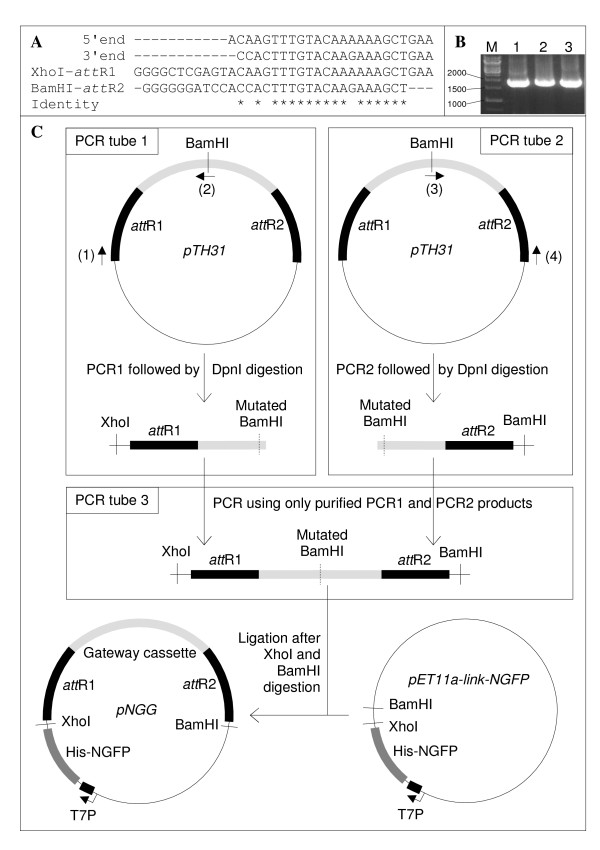
**Construction of pNGG**. (**A**) Alignment of (from top to bottom): the 5' and 3' ends of pTH31 Gateway^® ^cassette; XhoI-*att*R1 PCR primer; BamHI-*att*R2 PCR primer. Sequence identity is denoted by asterisks below the alignment. (**B**) The Gateway^® ^cassette was PCR amplified using pTH31 as template, and either primer XhoI-*att*R1 alone (lane 1), primer BamHI-*att*R2 alone (lane 2), or primers XhoI-*att*R1 and BamHI-*att*R2 (lane 3). Markers size is indicated on the left in base pairs. (**C**) "Two-halves" making of pNGG. The plasmids are not at scale. Light grey, Gateway cassette. Black, *att*R recombination sites. Primer (1), XhoI-*att*R1. Primer (2), BamHI-mut-R. Primer (3), BamHI-mut-F. Primer (4), BamHI-*att*R2 (Table 3).

Since neither pET11a-link-NGFP nor pMRBAD-link-CGFP are Gateway^® ^plasmids, as a first step in this project we modified pET11a-link-NGFP to make it a Gateway^® ^reporter expression plasmid. This conversion is generally performed by inserting a blunt-end synthetic cassette that is flanked by *att*R1 and *att*R2 recombination sites, and bears two constitutive genes: a chloramphenicol resistance (Cm^r^) gene, and a counter-selectable (*ccd*B) gene. The cassette can be purchased from Invitrogen in three reading frames, and is intended to be inserted in a blunt (or blunted) restriction site of the plasmid to modify. However, such a restriction site may not exist at the desired position, and blunt end cloning requires screening not only for cassette-containing plasmids but also for the correct orientation of the cassette with regard to the promoter. Since pET11a-link-NGFP cloning site contains two restriction sites (XhoI and BamHI), an alternative to the classical approach was to PCR amplify the cassette using whatever Gateway^® ^destination plasmid as template and primers hybridizing to the 5' and 3' ends of the cassette and flanked by XhoI and BamHI restriction sites, respectively. Unfortunately, because of the high homology between the 5' and 3' ends of the Gateway^® ^cassette (Figure 3A), each primer was found to be able to hybridize to both ends of the cassette (Figure 3B). To circumvent this problem, we devised the strategy depicted in Figure 3C. To prevent hybridization of the 5' primer with the 3'primer site and *vice versa*, the 5' and 3' halves of the cassette were PCR amplified separately (PCR tube 1 and PCR tube 2, Figure 3C) using the Gateway^® ^destination plasmid pTH31 [4] as template. This template contained a single BamHI restriction site in the middle of the cassette which had to be mutated before cloning. We took advantage of its central location to develop a pair of internal overlapping primers (primers 2 and 3) encoding a mutated BamHI site to mutate the internal BamHI restriction site during the amplification of the two cassette halves. Internal PCR primers 2 and 3 were 100% complementary to each other, so that the PCR products from tube 1 and tube 2 overlapped. After DpnI treatment to remove the template, and purification of the PCR products to remove PCR primers, a third PCR was run that only used an equimolar amount of the two PCR products, *i.e*., without adding external primers nor template (PCR tube 3, Figure 3C). This "elongation PCR" reconstituted the full-length cassette which was then ligated into pET11a-link-NGFP after BamHI and XhoI digestion. This Gateway^® ^reporter expression plasmid was called pNGG (plasmid N-GFP Gateway^®^) and is available upon request. Incidentally, this "two halves" strategy is generally applicable whenever direct directional cloning is hampered by a high homology of the two PCR primers.

**Table 3:** Effect of the molar ratios of *wt*N_TAIL_-PCR (donor) and non-linearized pNGG (acceptor construct).

	Donor	Acceptor construct	Number of clones
Electroporation	100 fmoles	25 fmoles	0.95 × 10^7^
	100 fmoles	100 fmoles	0.4 × 10^7^

Heat shock	100 fmoles	25 fmoles	0.47 × 10^5^
	100 fmoles	100 fmoles	0.16 × 10^5^

The number of *E. coli *colonies provided by using two molar ratios of donor and acceptor construct in a 5 μl LR reaction is reported. T7pRos *E. coli *cells were treated as described in Table 1

In a first step, the efficiency of LR reactions using different donors and acceptors was evaluated under different experimental conditions. We first assessed the efficiency of an LR reaction that made use of a non-mutated linear molecular species bearing *att*L recombination sites at the extremities (*wt*N_TAIL_-PCR, which could be described as a "ghost pENTR clone" since it is devoid of most of the pENTR backbone) and a circular Gateway^® ^reporter expression plasmid (non-linearized pNGG), and compared it to that of LR reactions that were carried out with different combinations of linear and non-linear substrates. The results are reported in Table 4. The combination used in the strategy we devised, *i.e. wt*N_TAIL_-PCR and non-linearized pNGG (Table 4, line 3), provided at least as good results as the other combinations, in particular when compared to the reference *wt*N_TAIL_-pDONR/non-linearized pNGG combination (Table 4, line 1). Thus, a linear DNA flanked by full-length *att*L recombination sites proved to be an efficient substrate for the LR reaction. We also checked whether the use of non-equimolar ratios of either LR reaction substrates, as suggested by Invitrogen, was optimal. The results, reported in Table 3, indicated that 100 fmoles of the donor construct and 25 fmoles of the acceptor construct provided at least as good results as an equimolar amount (100 fmoles of each), suggesting that 25 fmoles of non-linearized pNGG did not limit the LR reaction efficiency under these experimental conditions. The strategy we devised relies on the use of a pair of generic *att*L primers to generate an epPCR product flanked by the full-length *att*L1 and *att*L2 recombination sites (Stage 1 in the right flowchart of Figure 1). Since each *att*L recombination site is 100 bp long (Additional file 1: Figure S1), a risk potentially existed that mutations had been introduced by Mutazyme II in these sites during epPCR elongation steps. This would lead to an epPCR product partly unsuitable for the LR reaction, thereby ultimately resulting in a library complexity drop. As one can see in the first two lines of Table 5, 100 and even 10 fmoles of N_TAIL_-epPCR product provided at least as many clones as did 100 fmoles of N_TAIL_-PCR product generated by a proof-reading Taq polymerase (Table 4 third line, and Table 3 first line). This suggests that if mutations occurred in the *att*L recombination sites during the epPCR, they did not interfere with the downstream LR reaction. This result is not completely surprising since it has been shown that deleting as much as 50% of *att*L recombinant site did not significantly reduce the percentage of recombinant clones [23]. Finally, we investigated the reproducibility of our approach. As shown in Table 5 (last three lines), the results obtained with N_TAIL_/pNGG were not unique to this system, as comparable results were obtained when coding sequences with a composition and length (LadS, PA3059) different from that of N_TAIL _were used in LR reactions with another reporter expression plasmid (pTH31). This suggested that using a linear DNA generated by epPCR flanked by *att*L sites, and a non-linearized plasmid in the LR reaction was a generally applicable method.

**Table 4:** Comparison of expression library complexities provided by different combinations of linear and non-linear LR substrates using *wt*N_TAIL _sequence.

Donor	Acceptor construct	Number of clones
*wt*N_TAIL_-pDONR^1^	Non-linearized pNGG	0.3 × 10^7^
*wt*N_TAIL_-pDONR^1^	SalI-linearized pNGG	0.4 × 10^7^
*wt*N_TAIL_-PCR^2^	Non-linearized pNGG	1.6 × 10^7^
*wt*N_TAIL_-PCR^2^	SalI-linearized pNGG	0.25 × 10^7^

The number of *E. coli *colonies provided by various combinations of 100 fmoles of donor and of 25 fmoles of acceptor construct in a 5 μl LR reaction is reported. T7pRos *E. coli *cells were electroporated and spread on ACplates

^1^*wt*N_TAIL _coding sequence borne by pDONR. ^2^*wt*N_TAIL _coding sequence amplified using *att*L1 and *att*L2 primers

**Table 5:** Comparison of expression library complexities provided by different combinations of epPCR products and of non-linearized acceptor constructs

epPCR product	Non-linearized acceptor construct	Number of clones
N_TAIL_^1 ^10 fmoles	pNGG^4^	2.8 × 10^7^
N_TAIL_^1 ^100 fmoles	pNGG^4^	3.25 × 10^7^
LadS^2 ^10 fmoles	pTH31^5^	6.65 × 10^7^
LadS^2 ^100 fmoles	pTH31^5^	3.85 × 10^7^
PA3059^3 ^42 fmoles	pTH31^5^	1 × 10^7^

The number of *E. coli *colonies provided by various combinations of epPCR product and of 25 fmoles of acceptor constructs in 5 μl of LR reaction is reported. T7pRos *E. coli *cells were electroporated and spread on ACplates

^1^N_TAIL _PCR product is 661 bp. ^2^LadS PCR product is 696 bp. ^3^PA3059 PCR product is 1,780 bp. These three PCR product lengths include the *att*L1 and *att*L2 recombination sites. ^4^pNGG is 8,012 bp before LR recombination. ^5^pTH31 is 8,306 bp before LR recombination.

**Figure 4:**
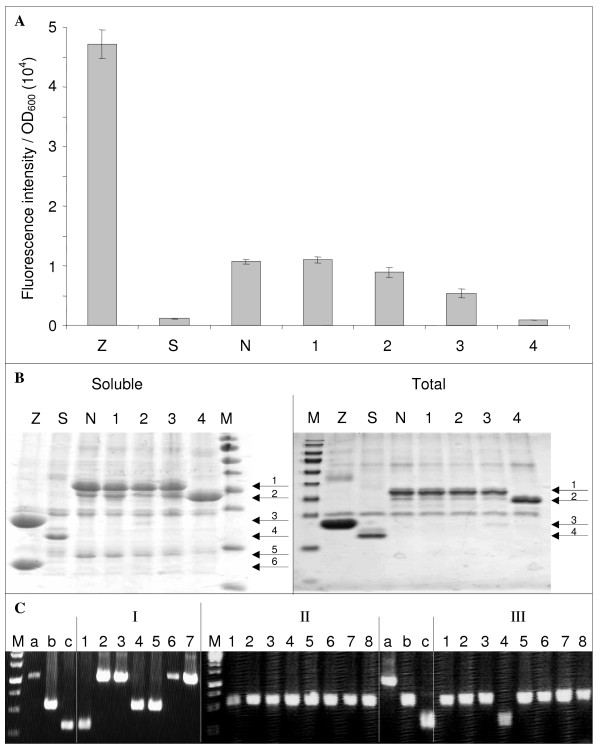
**Representative results of library screening and sub-cloning experiments**. (**A**) The fluorescence to OD_600 _ratio (mean value and standard deviation of a triplicate experiment) of the clones indicated on the × axis were determined as described in Methods. Z, Leucine zippers; S, Stop-N_TAIL_; N, *wt*N_TAIL_; 1-3, full length mutated N_TAIL_; 4, truncated N_TAIL _mutant. (**B**) His-tagged proteins expressed by clones Z, S, N, 1-4 (Figure 4A) were purified by affinity chromatography on IMAC as described in Methods, and were analyzed by SDS-PAGE using 15% polyacrylamide gels and Coomassie blue staining. Soluble, His-tagged proteins were purified under non denaturing conditions from the soluble fraction of the *E. coli *lysate. Total, His-tagged proteins were purified under denaturing conditions from total *E. coli *lysate. Soluble and total fractions were obtained from a duplicate culture. M, molecular size markers (from top to bottom: 170, 130, 100, 70, 55, 40, 35, 25, 15, 10 kDa). Arrows indicate the different purified proteins: 1, NGFP- *wt*N_TAIL _and NGFP-full-length N_TAIL _variants (34 kDa); 2, NGFP-truncated N_TAIL _variant 4 (29.4 kDa); 3, NGP-Z (22.8 kDa); 4, NGFP (*i.e*., Stop-N_TAIL_) (20.4 kDa); 5, XD-CGFP (15.5 kDa); 6, Z-CGFP (13.3 kDa). (**C**) PCR screening of mutated N_TAIL _sub-cloning experiment from pNGG to pDEST17O/I. PCR control a, 1,046 bp; b, 560 bp; c, 333 bp. PCR screening II and III were run in the same gel along with controls a, b, and c. M, molecular size markers (from top to bottom: 2000, 1500, 1000, 750, 500, 250 bp).

In the next step, we evaluated the ability of our method for generating epPCR libraries (Stage 1 in the right flowchart of Figure 1) to provide mutants of interest. To that end, an epPCR library of N_TAIL _in pNGG (Table 5, second line) was screened for clones displaying higher or lower fluorescence with respect to that of *wt*N_TAIL _when co-expressed with XD as described in Methods. More than 300 clones complying with these criteria were manually selected and characterized in terms of their fluorescence and of their sequence (Gruet *et al*., unpublished results). The latter revealed an average mutation rate of 1% base pairs. Attempts to increase this rate by performing additional epPCRs failed for the reasons explained in Additional file 2: Figure S2. Figure 4A shows the fluorescence of a representative sample of the selected clones. Antiparallel leucine zippers (Z) (pET11a-Z-NGFP and pMRBAD-Z-CGFP) were used as positive control of interaction [18] because of their high affinity, expression, and solubility. An N_TAIL _coding sequence with an in-frame stop codon located just downstream *att*B1 was used as negative control (Stop-N_TAIL_, S). This construct expresses only the NGFP moiety which is unable to interact with XD-CGFP. The reference fluorescence value was provided by *wt*N_TAIL _(N). A representative set of 4 mutants (1-4) with a fluorescence similar to or lower than that of *wt*N_TAIL _is reported. The relationship between the mutations borne by these mutants (Additional file 3: Text S1) and their specific fluorescence is beyond the scope of this study and will be discussed elsewhere (Gruet *et al*., unpublished results). Notably, clone 4 featured a stop codon (R489 (CGA) > (TGA) Stop) that resulted in a truncated form of N_TAIL_. Interestingly, this deletion perfectly mimics an already published variant (N_TAILΔ2,3_) that had been shown to display a considerably lower (two orders of magnitude) affinity towards XD [10]. Since we could not rule out *a priori *that a decreased fluorescence could be due to decreased protein expression and not to decreased N_TAIL_-NGFP/XD-CGFP interaction, we analyzed the total and soluble fractions of the different N_TAIL_-NGFP clones (S, N, 1-4). Taking advantage of the presence of a 6His tag appended at the N-terminus of NGFP [18], the His-tagged proteins expressed by clones Z, S, N, and 1-4 were purified by immobilized metal (Ni^2+^) affinity chromatography (IMAC) from the total *E coli *lysate under denaturing conditions, or from the soluble fraction of this latter under non denaturing conditions. The eluants from IMAC were analyzed by SDS-PAGE (Figure 4B). Notably, Stop-N_TAIL_, *wt*N_TAIL_, and all four mutants exhibited comparable levels of total expression of the NGFP-N_TAIL _fusion protein (arrows 1 and 2 in Figure 4B) suggesting that fluorescence differences were due to different interaction abilities and not to different protein expression levels. Moreover, the expression level of the NGFP-N_TAIL _protein by the non-fluorescent mutant 4 was even slightly higher than that of the other mutants (arrow 2 in Figure 4B). Under non denaturing conditions, the interacting partner fused to CGFP and devoid of His-tag (arrows 5 and 6 in Figure 4B) was co-purified with the His-tagged protein. As expected, its amount paralleled the fluorescence intensities reported in Figure 4A. By contrast, when proteins were purified by IMAC under denaturing conditions, only the His-tagged moiety was recovered from total *E coli *lysate. Altogether, these results indicate that different N_TAIL_-XD interaction capabilities due to specific mutations in the N_TAIL _sequence and not different N_TAIL _expression levels accounted for the different fluorescence intensities reported in Figure 4A. Thus, our one step method for generating epPCR libraries (Stage 1 in the right flowchart of Figure 1) proves to be an effective mean to yield mutants of interest.

In view of investigating interaction capabilities in the absence of the GFP moiety (to be published elsewhere), the mutated N_TAIL _coding sequences were sub-cloned from pNGG (reporter expression plasmid) to pDEST17O/I (non-reporter expression plasmid), a frame-compatible plasmid allowing proteins to be expressed with a simple N-terminal His-tag [24]. Sub-cloning was performed as described above (Figure 1, stage 2, right panel and Figure 2). The efficiency of this approach is highly dependent on the efficiency of DpnI treatment performed after each PCR. An inefficient DpnI treatment after PCR1 would generate a background made of the donor construct (mutant sequence in pNGG). An inefficient DpnI treatment after PCR2 would generate a background made of the acceptor construct (*wt *sequence in pDEST17O/I). Unfortunately, this signal-to-noise ratio could be appraised only by sequencing plasmids borne by several randomly chosen clones. To avoid sequencing PCR1 or PCR2 background, we introduced a screening step so as to be able to distinguish background plasmids from the desired construct. The easiest way to do that was to use a pair of screening primers that amplify fragments of different lengths from the three possible constructs (*i.e*., the correct construct, and PCR1 and PCR2 backgrounds). Although a primer pair made of T7prom and *att*B2 primers could distinguish pNGG from pDEST17O/I, it failed to differentiate a mutated N_TAIL _from the *wt*N_TAIL _sequence in pDEST17O/I, as both have the same length. A solution to this issue was to use as template in PCR2 an internally deleted *wt*N_TAIL _sequence (Figure 2) instead of the full-length sequence as envisaged by MEGAWHOP [21]. The internally deleted (227 bp deletion) N_TAIL _borne by pDEST17O/I (pDEST17O/I-idN_TAIL_, Table 2) was constructed by inverse PCR using the pDEST17O/I-N_TAIL _plasmid (Table 2) as template and primers p17O/ISal1F and p17O/ISal1R (Table 6). Following DpnI digestion, the PCR product was purified and then digested with SalI and self-ligated (Figure 2). Under these conditions, PCR screening *E. coli *colonies after transformation with DpnI-treated PCR2 product using T7prom and attB2 primers was expected to lead to amplification of either a 1,046 bp band (PCR1 background), or a 560 bp band (correct construct), or a 333 bp band (PCR2 background). Typical PCR screening results are reported in Figure 4C. First experiments (experiment I in Figure 4C) revealed a low signal (*i.e*., correct construct) to background ratio. In gel I, clone 1 was PCR2 background, while clones 2, 3, 6 and 7 were PCR1 background and only clones 4 and 5 contained the correct construct. The high background (5 clones out of 7) denoted inefficient DpnI treatments, particularly after PCR1 (4 clones out of 7). This issue was addressed in two different ways. Firstly, we reduced the background by improving DpnI treatment and by reducing the amount of the template used in PCR1 as the PCR template is responsible for the background. Secondly, as the signal is proportional to (and limited by) the amount of megaprimers used in PCR2 we increased the signal by increasing the amount of megaprimers produced by PCR1. In practice, to both increase the signal and decrease the background we combined several modifications of the reaction: more DpnI was used for longer incubation times, more of each primer, more PCR cycles, larger PCR volumes and less PCR template were used in experiments II and III (Additional file 4: Table S1) to generate the megaprimers (PCR1). In experiment II, the template used for PCR2 was full-length N_TAIL _in pDEST17O/I. The new experimental conditions (Additional file 4: Table S1) completely abolished PCR1 background (no band detectable at 1,046 bp in Figure 4C, experiment II). However, the use of full-length N_TAIL _in pDEST17O/I as template in PCR2 did not allow detecting PCR2 background. Therefore, an experiment was performed as in experiment II except that internally deleted N_TAIL _in pDEST17O/I was used as template in PCR2. The results (experiment III in Figure 4C) confirmed the absence of PCR1 background and pointed out a low level (similar to that of experiment I although PCR2 template had been increased, see Additional file 4: Table S1) of PCR2 background. PCR-positive clones 1, 2, 3 and 5 from experiment III were sequenced, and proved to bear the correct insert in the correct plasmid. In addition, sequencing ruled out the presence of any unwanted mutations that could have been introduced because of the increase (from 10 to 30) in the number of cycles in PCR1.

**Table 6:** Primers used in this study

Name	Sequence	Purpose
Nco1-MeV.XD	GGGGCCATGGGCGCATCACGCAGTGTAATCCGCTCC	XD PCR amplification
MeV.XD-AatII	GGGGGACGTCGACTTCATTATTATCTTCACCAGCAT	

N_TAIL_F	GGGGACAAGTTTGTACAAAAAAGCAGGCTCTACTACTGAGGACAAGATCAGTAGA	N_TAIL _PCR amplification
N_TAIL_R	AGCTTTCTTGTACAAAGTGGTGGATCCCCCC	

StopNtail	GGGGACAAGTTTGTACAAAAAAGCAGGCTCTTAATAAACTACTGAGGACAAGATCAGTAGA	StopNtail PCR amplification

XhoI-*att*R1 (primer 1)	GGGGCTCGAGTACAAGTTTGTACAAAAAAGCTGAA	pNGG construction (Gateway^® ^cassette 5' halve)
BamHI-mut-R (primer 2)	TCTGGCTTTTAGTAAGCCGGAACCTCTAGATTACGCCCCGCCCTG	

BamHI-mut-F (primer 3)	CAGGGCGGGGCGTAATCTAGAGGTTCCGGCTTACTAAAAGCCAGA	pNGG construction (Gateway^® ^cassette 3' halve)
BamHI-*att*R2 (primer 4)	GGGGGGATCCACCACTTTGTACAAGAAAGCT	

p17O/ISal1F	GGGGGTCGACGCAGGAATCTCGGAAGAACAAGGC	PCR construction of pDEST17O/I-idN_TAIL_
p17O/ISal1R	GGGGGTCGACCGTGTAGAAATGATACTTGGGC	

T7prom	TAATACGACTCACTATAGG	Sub-cloning screening
*att*B2	CCACTTTGTACAAGAAAGCTGGGT	

*att*L1	TCGCGTTAACGCTAGCATGGATCTC	epPCR
*att*L2	GTAACATCAGAGATTTTGAGACAC	

## Discussion

In this study, we described a new method for creating high complexity epPCR libraries based on a modified use of the Gateway^® ^recombination cloning technology. Compared to the conventional Gateway^® ^protocol ([4] and stage 1 in the left flowchart of Figure 1), our method is faster as it skips some steps such as the BP reaction and the associated *E. coli *transformation and plasmid purification. Note that although we only used epPCR for generating random mutant libraries, we believe that our strategy could be easily applied to the generation of libraries based on DNA shuffling [2]. Since the conventional sub-cloning of selected mutated sequences requires the same steps as those typically used to create the library ([4] and stage 2 in the left flowchart of Figure 1), we also devised a sub-cloning strategy that allows several mutant sequences to be sub-cloned in parallel from the reporter expression plasmid used to screen the library to another expression plasmid devoid of the screening tag (GFP in this case). We have applied and validated the method in three directed evolution projects, and provided here the first results obtained in the case of the N_TAIL_-XD system. In the other two projects, we constructed other epPCR libraries that made use of other target sequences to evolve, as well as of another reporter expression plasmid. In all cases, comparable library complexities were obtained (Table 5 and unpublished data).

As shown in Table 1, skipping the BP reaction used in the standard protocol to clone the epPCR product in pDONR (stage 1 in Figure 1) appears to preserve the library complexity. However, one may argue that the price paid for this preservation is that the *wt *sequence must first be cloned into pDONR, and so the benefit of our strategy (*i.e*. the reduction in the number of steps) would be abrogated by this first mandatory "pre-cloning" BP reaction. We would like to point out that this is not the case. This starting construct is instrumental not only in the epPCR step but also, directly or indirectly, in three other steps of our procedure (see dashed line in the right flowchart of Figure 1). Firstly, this pDONR construct bearing the *wt *sequence is required to generate by LR reaction the pDEST17O/I-*wt*N_TAIL _construct (*i.e*. the non-reporter expression plasmid bearing the *wt *sequence) that will be used as a control in comparative expression experiments with the selected mutated sequences (Figure 1). Secondly, pDEST17O/I-*wt*N_TAIL _allows the construction of the internally deleted template used in the sub-cloning (Figure 2). Thirdly, pDEST17O/I-*wt*N_TAIL _also provides a positive control in PCR screenings aimed at avoiding the sequencing of background clones (Figure 4C). By contrast, since the standard strategy (stage 1 in the left flowchart of Figure 1) used a non Gateway^® ^plasmid as template for the epPCR, the same result will require much more work and steps (compare dashed lines in the left and right flowcharts of Figure 1). Thus, what could first appear as a constraint finally proves to be a saving of time when compared to the standard protocol.

RF cloning and MEGAWHOP could not be used in our sub-cloning protocol exactly as they were published, and needed to be adapted for the following reasons. In the published RF cloning technique [22], PCR1 was performed using genomic DNA as template, and so inefficient DpnI digestion would simply result in bacteria being transformed with genomic DNA. Since the latter did not provide any antibiotic resistance it could not generate any background, whereas the template was responsible for PCR1 background in our case (Figure 4C). In the case of MEGAWHOP [21], donor and acceptor plasmids were the same. Here again, inefficient DpnI digestion of PCR2 product would only increase the amount of non mutated sequences in the final epPCR library, a situation frequently encountered even at high mutagenesis rates (A. Gruet, unpublished observations), whereas it was responsible for PCR2 background in our case (Figure 4C).

It is noteworthy that the sub-cloning method that we developed was made possible because of the following two features. Firstly, donor (pNGG) and acceptor (pDEST17O/I) plasmids were both Gateway^® ^plasmids and hence shared common 5' and 3' PCR priming sites. Moreover, these PCR priming sites did not require specific but generic *att*B1 and *att*B2 primers. Secondly, thanks to the specific founding features of Gateway^®^, pNGG and pDEST17O/I were 5' and 3' reading frame compatible, a feature that can be easily obtained for any expression plasmid suitably modified to bear the Gateway^® ^cassette. For example, we have taken advantage of this feature to sub-clone mutated coding sequences from pTH31 to pTH24 [4] in another directed evolution project (B. Coutard, unpublished results).

Although it does simplify sub-cloning when more than five mutant coding sequences have to be processed in parallel, the sub-cloning strategy that we devised could be further optimized by using a PCR2 template that could be detected directly on plates. This would save the time devoted to pick up and grow randomly chosen clones and to purify and screen their plasmids so as to distinguish them from those from background colonies. Actually, we sought at using Rubredoxin [25] as such a possible phenotypic marker of PCR2 background. Unfortunately however, following transformation of *E. coli *with a pDEST17O/I plasmid bearing the sequence of a synthetic *Thermotoga maritima *Rubredoxin gene (NCBI reference sequence: NP_228468.1) that had been optimized for translation in *E. coli*, no red colonies were obtained (data not shown). Another even more stringent mean would be to use a *ccd*B constitutive gene in pDEST17O/I instead of an internally deleted N_TAIL _[26]. In that case, there would be no PCR2 background as clones bearing the *ccd*B gene are not viable.

Before concluding, we would like to point out that the method described here is proposed as a toolbox made of three independent parts:* (i)* the production of epPCR libraries, *(ii)* the "two halves" construction of a Gateway^® ^plasmid, *(iii)* a sub-cloning strategy. The N_TAIL_-XD project provided an opportunity to describe a combined usage of the three parts, but these can be used separately (B. Coutard, unpublished results).

The Gateway^® ^cloning technology has been a tremendous breakthrough since it was implemented in the early 2000s. Perhaps not by chance, its commercial availability happened to coincide with the beginning of the Structural Genomics era. This coincidence undoubtedly helped Structural Genomics programs to comply with their time schedule [1]. To our surprise, we have been unable to find numerous directed evolution projects that made use of the Gateway^® ^technology. We hope that the modified protocol described in this study will contribute to a broader usage of this powerful technology in studies aimed at evolving proteins.

## Conclusions

We have described a method to create high complexity epPRC libraries using only the LR reaction of the Gateway^® ^cloning technology. Directly cloning an epPCR product in the plasmid used for screening the library allows eliminating not only the BP reaction but also the associated *E. coli *cell transformation and plasmid purification required by the usual Gateway^® ^procedure. Thus, the same result can be obtained with half the amount of work.

## Methods

### Materials

Turbo broth (TB) was from AthenaES™. NucleoSpin^® ^Extract II and NucleoSpin^® ^Plasmid kit (Macherey-Nagel) were used for purifying PCR products and for plasmid mini-preparations, respectively. DNA ligations were performed using Quick Ligation Kit (Roche). GeneMorph^® ^II Random Mutagenesis Kit (Stratagene) was used for epPCR experiments. Preparative and analytical PCRs were carried out using *Pfx *and Taq polymerase (Invitrogen), respectively. All primers were purchased from Operon. DNA sequencing was performed by GATC Biotech.

### DNA constructs

pMRBAD-link-CGFP [19], encoding the C-terminal half (aa 158-238) of eGFP under the control of the P_BAD _promoter (inducible by L-arabinose) and allowing upstream in frame cloning of DNA fragments, was a kind gift of Lynne Regan, as were pET11a-link-NGFP, pET11a-Z-NGFP and pMRBAD-Z-CGFP. These latter two encode fusion proteins between leucine zippers and the two halves of the eGFP, while pET11a-link-NGFP encodes the N-terminal half (aa 1-157) of eGFP under the control of the T7 promoter (inducible by IPTG) and allows downstream in-frame cloning of DNA fragments [19]. pMRBAD-XD-CGFP was constructed as follows. The coding sequence of XD was PCR amplified using primers Nco1-MeV.XD and MeV.XD-AatII (Table 6) and a plasmid encoding the X domain (aa 459-507) of the measles virus phosphoprotein (strain Edmonston B) (pDEST14/XD_HC_) as template [9]. DpnI-treated purified PCR product and pMRBAD-link-CGFP were ligated after NcoI and AatII digestion. pDONR-N_TAIL _was obtained by BP reaction with a PCR N_TAIL _fragment amplified using primers N_TAIL_F and N_TAIL_R (Table 6), and a plasmid encoding the measles virus nucleoprotein (strain Edmonston B) (pET-21a/N) as template [27]. pDONR-Stop-N_TAIL _was obtained by BP reaction with a PCR N_TAIL _fragment amplified using primers StopNtail and N_TAIL_R (Table 6) and pDONR-N_TAIL _as template. StopNtail has two Stop codons before the first N_TAIL _codon. pNGG-N_TAIL _and pNGG-Stop-N_TAIL _were obtained by LR reaction using pNGG as destination vector and pDONR-N_TAIL _or pDONR-Stop-N_TAIL _as donor constructs, respectively. pDEST17O/I-N_TAIL _was obtained by LR reaction using pDONR-N_TAIL _and pDEST17O/I [24]. The constructs were verified by sequencing and found to conform to expectations. The plasmids used in this study are summarized in Table 2.

### Strains and electroporation

DB3.1 cells (Invitrogen) were used to propagate non recombined Gateway^® ^plasmids, and TAM1 cells (Active Motif) to propagate non-Gateway^® ^and recombined Gateway^® ^plasmids. T7 cells (New England Biolabs) bearing the pLysS plasmid from Rosetta(DE3)pLysS cell (Novagen) (referred to as T7pRos) were used for protein expression and for epPCR library screening.

Electrocompetent T7pRos cells were prepared as follows. Frozen cells that had been previously transformed with pMRBAD-XD-CGFP were used to seed 1 L of LB containing 50 μg/ml kanamycin and 34 μg/ml chloramphenicol, and allowed to grow until OD_600 _= 0.5. The cells were recovered by centrifugation for 10 min at 5000 g, and resuspended in 400 ml of ice-cold water. Three additional washings were performed using 300 and then 200 ml of ice-cold water. The cells were then washed with 80 ml of ice-cold 10% glycerol, and finally re-suspended in 4 ml of the same buffer. Aliquots of 1 ml were stored frozen at -80°C until use. For electroporation, 50 μl of cells were mixed with 1 μl of DNA and electroporated in 1 mm wide cuvettes (Eurogentec CE-0001-50) at 1660 V using an Eppendorf electroporator 2510. Under these conditions, an average time constant of ~5.4 milliseconds was observed.

### Error-prone PCR and Gateway^® ^cloning

Error-prone PCR experiments were performed following the indications provided by the GeneMorph^® ^II Random Mutagenesis Kit instruction manual (Stratagene) using 10 ng of template in 50 μl of PCR mix. At the end of the PCR, 1 μl (20 U) of DpnI (New-England Biolabs) was added to the PCR mix which was then incubated for 1 h at 37°C. After purification, the quality and quantity of the PCR product were estimated as described in the kit manual and by spectrophotometry.

LR reactions were performed overnight at 26°C in a dry incubator in a final volume of 5 μl containing 1.5 μl of each of the donor and acceptor construct, 1 μl of 5× LR buffer, and 1 μl of LR clonase enzyme mix (Invitrogen). The next day, 1 μl of a 2 μg/μl proteinase K solution (Euromedex, EU0090) was added and the reaction mix was incubated for 15 min at 37°C. LR reactions were diluted to 50 μl with water, and 1 μl of this dilution was used to electroporate T7pRos cells as described above. Immediately after electroporation, the cells were resuspended in 15 ml of SOC medium, and then incubated for 1 hour at 37°C under 200 rpm shaking. Serial dilutions of an aliquot were plated on AKCplate to assess the complexity of the library. The remaining bacterial suspension was supplemented with ampicillin, kanamycin and chloramphenicol at a final concentration of 100, 50 and 34 μg/ml, respectively, and grown at 37°C under 200 rpm shaking. When the culture medium became turbid (~5 h), it was supplemented with glycerol at a final concentration of 20% (volume/volume), and 300 μl aliquots were frozen at -80°C.

### Library screening

An aliquot of the library was thawed and serial dilutions were spread on AKCplates to assess the number of clones. An aliquot from the same tube was then spread on AKCplates at a cell density allowing isolated colonies to be obtained after overnight growth at 37°C. Colonies were randomly picked up and individually grown at 37°C overnight under 700 rpm shaking in 500 μl of TB containing 100 μg/ml ampicillin, 50 μg/ml kanamycin and 34 μg/ml chloramphenicol (TBAKC) in a 96-well deep-well plate. The next day, another 96-well deep-well plate containing the same volume of the same culture medium was seeded with 50 μl of the overnight culture, and the remaining pre-culture was used to individually seed 96-well ampicillin agar plates provided by GATC Biotech and sent to the same company for plasmid purification and sequencing. The freshly seeded deep-well plate was grown for 1 h at 37°C under 700 rpm shaking. IPTG and arabinose were then added at final concentrations of 0.5 mM and 2%, respectively, and protein expression was allowed to proceed at 17°C for at least 20 h. Culture medium, culture temperature during protein expression, and IPTG and arabinose concentrations leading to the best signal-to-noise ratio were determined by using a fractional factorial approach modified from [28], the detail of which are provided in Additional file 5: Figure S3. At the end of the culture, the deep-well plate was spun for 3 min at 1,500 g and the culture medium discarded. The cell pellets were re-suspended in 500 μl of PBS by shaking at 700 rpm for 20 min at 17°C. The fluorescence of 100 μl of re-suspended cells was measured using a TECAN GENios *Plus *spectrofluorimeter. The cell density was determined by measuring the optical density at 600 nm (OD_600_) of 100 μl of re-suspended cells diluted 10 times with PBS, using the same spectrofluorimeter. The results were expressed as the fluorescence to OD_600 _ratio.

### Protein expression and purification

T7pRos cells were extemporaneously co-transformed by heat-shock with the following pairs of constructs: pMRBAD-XD-CGFP and either pNGG-N_TAIL _(positive control N) or pNGG-Stop-N_TAIL _(negative control S) or pNGG-N_TAIL _constructs bearing the mutated N_TAIL _sequences of interest (clones 1-4); pMRBAD-Z-CGFP and pET11a-Z-NGFP (positive fluorescence control Z). In all cases, transformants were selected on AKCplates. TBAKC (4 ml per well in a 24-well deep-well plate) was seeded with a single colony from AKCplates, and then incubated overnight at 37°C under shaking. The next day, 4 ml of TBAKC in 24-well deep-well plate were seeded with 200 μl of the overnight culture and shaken at 37°C at 200 rpm until the medium became turbid (from ~0.5 to ~1 OD_600_). IPTG and arabinose were added at final concentrations of 0.5 mM and 2%, respectively, and the cultures were incubated overnight at 17°C under 200 rpm agitation. Cells were then recovered by spinning the deep-well plate for 5 min at 3000 g. Each cell pellet was re-suspended in 1 ml of 50 mM Tris/HCl pH 8, 0.3 M NaCl, 0.1% Triton X100, 1 mM EDTA, 10 mM imidazole, 1 mM PMSF and 0.25 mg/ml lysozyme and frozen. After thawing, DNAseI and MgSO_4 _were added at final concentrations of 10 μg/ml and 20 mM, respectively and incubated at 37°C in a shaking incubator for 30 min. The deep-well plate was spun for 10 min at 3,000 g at 4°C. The supernatant (soluble fraction) was transferred to 1.5 ml microtubes and supplemented with 50 μl of a 50% (volume/volume) suspension of IMAC sepharose high performance (GE healthcare). The mixture was rotated for one hour on a wheel at 4°C. Sepharose beads were then washed three times with 1 ml of 50 mM Tris/HCl pH 8, 0.3 M NaCl, 50 mM imidazole, and bound His-tagged proteins were eluted with 100 μl of 50 mM Tris/HCl pH 8, 0.3 M NaCl, 500 mM imidazole. When purifying His-tagged proteins under denaturing conditions, the soluble and insoluble fractions were not separated by centrifugation after DNAseI treatment but the total lysate was directly supplemented with two volumes of 50 mM Tris/HCl pH 8, 8 M guanidinium chloride, 0.3 M NaCl, 10 mM imidazole, and with 100 μl of a 50% (volume/volume) suspension of IMAC sepharose beads. Subsequent steps were carried out as described for the non denaturing conditions, except that the experiment was performed at room temperature, and that washing and elution buffers were supplemented with 8 M urea. In all cases, eluted proteins were analyzed by SDS-PAGE.

## Abbreviations

Aa: Amino-acid; ACplates: LB agar 100 mm plates containing 100 μg/ml ampicillin and 34 μg/ml chloramphenicol; AKCplates: LB agar 100 mm plates containing 100 μg/ml ampicillin 34 μg/ml chloramphenicol and 50 μg/ml kanamycin; IPTG: Isopropyl β-D-1-thiogalactopyranoside; SDS-PAGE: Sodium dodecyl sulfate-polyacrylamide gel electrophoresis; TB: Turbo broth.

## Competing interests

The authors declare that they have no competing interests.

## Authors' contributions

AG obtained the original plasmids from Dr.LR, performed most of the experiments and participated in the writing of the manuscript. SL had the idea of using directed evolution for studying N_TAIL_-XD interaction, is the principal investigator of the ANR-08-PCVI-0020-01 program and participated in the writing of the manuscript. CB devised the method, participated in some experiments, directed the work of the first author and wrote the paper. All authors read and approved the final manuscript.

## Supplementary Material

Additional file 1 **Figure S1**. Recombination region of the pDONR201^® ^plasmid after BP reaction. Click here for file

Additional file 2 **Figure S2**. Attempts to increase the mutation rate above 1%. Click here for file

Additional file 3 **Text S1**. Nucleotide sequence of the coding sequences (S, N, 1-4) used in the experiments reported in Figure 4A and 4B. Click here for file

Additional file 4 **Table S1**. This table compares the experimental conditions of experiment I, II and III reported in Figure 4C. Click here for file

Additional file 5 **Figure S3**. Optimization of co-expression conditions. Click here for file

## References

[B1] Vincentelli R Bignon C Gruez A Canaan S Sulzenbacher G Tegoni M Campanacci V Cambillau C Medium-scale structural genomics: strategies for protein expression and crystallization Acc Chem Res 2003 36 3 165 172 10.1021/ar010130s 12641473

[B2] Stemmer WP DNA shuffling by random fragmentation and reassembly: in vitro recombination for molecular evolution Proc Natl Acad Sci USA 1994 91 22 10747 10751 10.1073/pnas.91.22.10747 7938023 PMC45099

[B3] Waldo GS Standish BM Berendzen J Terwilliger TC Rapid protein-folding assay using green fluorescent protein Nat Biotechnol 1999 17 7 691 695 10.1038/10904 10404163

[B4] van den Berg S Lofdahl PA Hard T Berglund H Improved solubility of TEV protease by directed evolution J Biotechnol 2006 121 3 291 298 10.1016/j.jbiotec.2005.08.006 16150509

[B5] Heddle C Mazaleyrat SL Development of a screening platform for directed evolution using the reef coral fluorescent protein ZsGreen as a solubility reporter Protein Eng Des Sel 2007 20 7 327 337 10.1093/protein/gzm024 17584755

[B6] Cao S Siriwardana CL Kumimoto RW Holt BF Construction of high quality Gateway entry libraries and their application to yeast two-hybrid for the monocot model plant Brachypodium distachyon BMC Biotechnol 2011 11 53 10.1186/1472-6750-11-53 21595971 PMC3239850

[B7] Longhi S Receveur-Brechot V Karlin D Johansson K Darbon H Bhella D Yeo R Finet S Canard B The C-terminal domain of the measles virus nucleoprotein is intrinsically disordered and folds upon binding to the C-terminal moiety of the phosphoprotein J Biol Chem 2003 278 20 18638 18648 10.1074/jbc.M300518200 12621042

[B8] Bourhis J Johansson K Receveur-Bréchot V Oldfield CJ Dunker AK Canard B Longhi S The C-terminal domain of measles virus nucleoprotein belongs to the class of intrinsically disordered proteins that fold upon binding to their physiological partner Virus Res 2004 99 157 167 10.1016/j.virusres.2003.11.007 14749181

[B9] Johansson K Bourhis JM Campanacci V Cambillau C Canard B Longhi S Crystal structure of the measles virus phosphoprotein domain responsible for the induced folding of the C-terminal domain of the nucleoprotein J Biol Chem 2003 278 45 44567 44573 10.1074/jbc.M308745200 12944395

[B10] Bourhis JM Receveur-Bréchot V Oglesbee M Zhang X Buccellato M Darbon H Canard B Finet S Longhi S The intrinsically disordered C-terminal domain of the measles virus nucleoprotein interacts with the C-terminal domain of the phosphoprotein via two distinct sites and remains predominantly unfolded Protein Sci 2005 14 1975 1992 10.1110/ps.051411805 16046624 PMC2279309

[B11] Morin B Bourhis JM Belle V Woudstra M Carrière F BGuigliarelli B Fournel A Longhi S Assessing induced folding of an intrinsically disordered protein by site-directed spin-labeling EPR spectroscopy J Phys Chem B 2006 110 41 20596 20608 10.1021/jp063708u 17034249

[B12] Belle V Rouger S Costanzo S Liquiere E Strancar J Guigliarelli B Fournel A Longhi S Mapping alpha-helical induced folding within the intrinsically disordered C-terminal domain of the measles virus nucleoprotein by site-directed spin-labeling EPR spectroscopy Proteins: Struct, Funct, Bioinf 2008 73 4 973 988 10.1002/prot.22125 18536007

[B13] Bernard C Gely S Bourhis JM Morelli X Longhi S Darbon H Interaction between the C-terminal domains of N and P proteins of measles virus investigated by NMR FEBS Lett 2009 583 7 1084 1089 10.1016/j.febslet.2009.03.004 19275899

[B14] Bischak CG Longhi S Snead DM Costanzo S Terrer E Londergan CH Probing structural transitions in the intrinsically disordered C-terminal domain of the measles virus nucleoprotein by vibrational spectroscopy of cyanylated cysteines Biophys J 2010 99 5 1676 1683 10.1016/j.bpj.2010.06.060 20816082 PMC2931715

[B15] Gely S Lowry DF Bernard C Ringkjobing-Jensen M Blackledge M Costanzo S Darbon H Daughdrill GW Longhi S Solution structure of the C-terminal × domain of the measles virus phosphoprotein and interaction with the intrinsically disordered C-terminal domain of the nucleoprotein J Mol Recognit 2010 23 435 447 10.1002/jmr.1010 20058326

[B16] Kavalenka A Urbancic I Belle V Rouger S Costanzo S Kure S Fournel A Longhi S Guigliarelli B Strancar J Conformational analysis of the partially disordered measles virus NTAIL-XD complex by SDSL EPR spectroscopy Biophys J 2010 98 6 1055 1064 10.1016/j.bpj.2009.11.036 20303863 PMC2849088

[B17] Ringkjøbing Jensen M Communie G Ribeiro ED Jr Martinez N Desfosses A Salmon L Mollica L Gabel F Jamin M Longhi S Intrinsic disorder in measles virus nucleocapsids Proc Natl Acad Sci USA 2011 108 24 9839 9844 10.1073/pnas.1103270108 21613569 PMC3116414

[B18] Magliery TJ Wilson CG Pan W Mishler D Ghosh I Hamilton AD Regan L Detecting protein-protein interactions with a green fluorescent protein fragment reassembly trap: scope and mechanism J Am Chem Soc 2005 127 1 146 157 10.1021/ja046699g 15631464

[B19] Wilson CG Magliery TJ Regan L Detecting protein-protein interactions with GFP-fragment reassembly Nat Methods 2004 1 3 255 262 10.1038/nmeth1204-255 16145770

[B20] Jackrel ME Cortajarena AL Liu TY Regan L Screening libraries to identify proteins with desired binding activities using a split-GFP reassembly assay ACS Chem Biol 2010 5 6 553 562 10.1021/cb900272j 20038141

[B21] Miyazaki K Takenouchi M Creating random mutagenesis libraries using megaprimer PCR of whole plasmid Biotechniques 2002 33 5 1033-1034-1036-1038 10.2144/02335st03 12449380

[B22] van den Ent F Lowe J RF cloning: a restriction-free method for inserting target genes into plasmids J Biochem Biophys Methods 2006 67 1 67 74 10.1016/j.jbbm.2005.12.008 16480772

[B23] Fu C Wehr DR Edwards J Hauge B Rapid one-step recombinational cloning Nucleic Acids Res 2008 36 9 e54 10.1093/nar/gkn167 18424799 PMC2396420

[B24] Vincentelli R Canaan S Campanacci V Valencia C Maurin D Frassinetti F Scappucini-Calvo L Bourne Y Cambillau C Bignon C High-throughput automated refolding screening of inclusion bodies Protein Sci 2004 13 10 2782 2792 15388864 10.1110/ps.04806004 PMC2286565

[B25] Kohli BM Ostermeier C A Rubredoxin based system for screening of protein expression conditions and on-line monitoring of the purification process Protein Expr Purif 2003 28 2 362 367 10.1016/S1046-5928(02)00704-0 12699702

[B26] Stech J Stech O Herwig A Altmeppen H Hundt J Gohrbandt S Kreibich A Weber S Klenk HD Mettenleiter TC Rapid and reliable universal cloning of influenza A virus genes by target-primed plasmid amplification Nucleic Acids Res 2008 36 21 e139 10.1093/nar/gkn646 18832366 PMC2588516

[B27] Karlin D Longhi S Canard B Substitution of two residues in the measles virus nucleoprotein results in an impaired self-association Virology 2002 302 2 420 432 10.1006/viro.2002.1634 12441086

[B28] Benoit I Coutard B Oubelaid R Asther M Bignon C Expression in Escherichia coli, refolding and crystallization of Aspergillus niger feruloyl esterase A using a serial factorial approach Protein Expr Purif 2007 55 1 166 174 10.1016/j.pep.2007.04.001 17533138

