# Supplementary material for: One-step generation of error-prone PCR libraries using Gateway® technology
Source: Microb Cell Fact. 2012 Jan 30;11:14. doi: 10.1186/1475-2859-11-14 (PMC3349575; doi:10.1186/1475-2859-11-14)
Supplement: Additional file 1 — Figure S1. Recombination region of the pDONR201® plasmid after BP reaction. [file 1475-2859-11-14-S1.PDF]

```
                                                          -
 251  CTTCCGACTG AGCCTTTCGT TTTATTTGAT GCCTGGCAGT TCCCTACTCT
      ----attL1 primer--------->     -----------------------
 301  CGCGTTAACG CTAGCATGGA TCTCGGGCCC CAAATAATGA TTTTATTTTG
      ------------attL1 recombination site------------------
 351  ACTGATAGTG ACCTGTTCGT TGCAACAAAT TGATGAGCAA TGCTTTTTTA
      ------------------------------------            ------
 401  TAATGCCAAG TTTGTAC AAA AAA GCA GGC TNN… GENE … NAC CCA
 ----------------- attL2 recombination site-----------------
 GCT TTC TTG TAC AAA GTG GGC ATTATAAGA AAGCATTGCT TATCAATTTG
      ---------------------------------------------------
2701  TTGCAACGAA CAGGTCACTA TCAGTCAAAA TAAAATCATT ATTTGCCATC
                         <-----attL2 primer---------
2751  CAGCTGCAGC TCTGGCCCGT GTCTCAAAAT CTCTGATGTT ACATTGCACA
```

Supplementary Figure S1. Recombination region of the pDONR201® plasmid after BP reaction. The hybridization sites of primers *att*L1 and *att*L2 on recombined pDONR201® are the “Forward- and Reverse-priming sites” in Invitrogen nomenclature (in blue), and are located upstream and downstream the *att*L1 (100 bp) and *att*L2 (100 bp) recombination sites (in red).
