# Supplementary material for: One-step generation of error-prone PCR libraries using Gateway® technology
Source: Microb Cell Fact. 2012 Jan 30;11:14. doi: 10.1186/1475-2859-11-14 (PMC3349575; doi:10.1186/1475-2859-11-14)
Supplement: Additional file 2 — Figure S2. Attempts to increase the mutation rate above 1%. [file 1475-2859-11-14-S2.PDF]

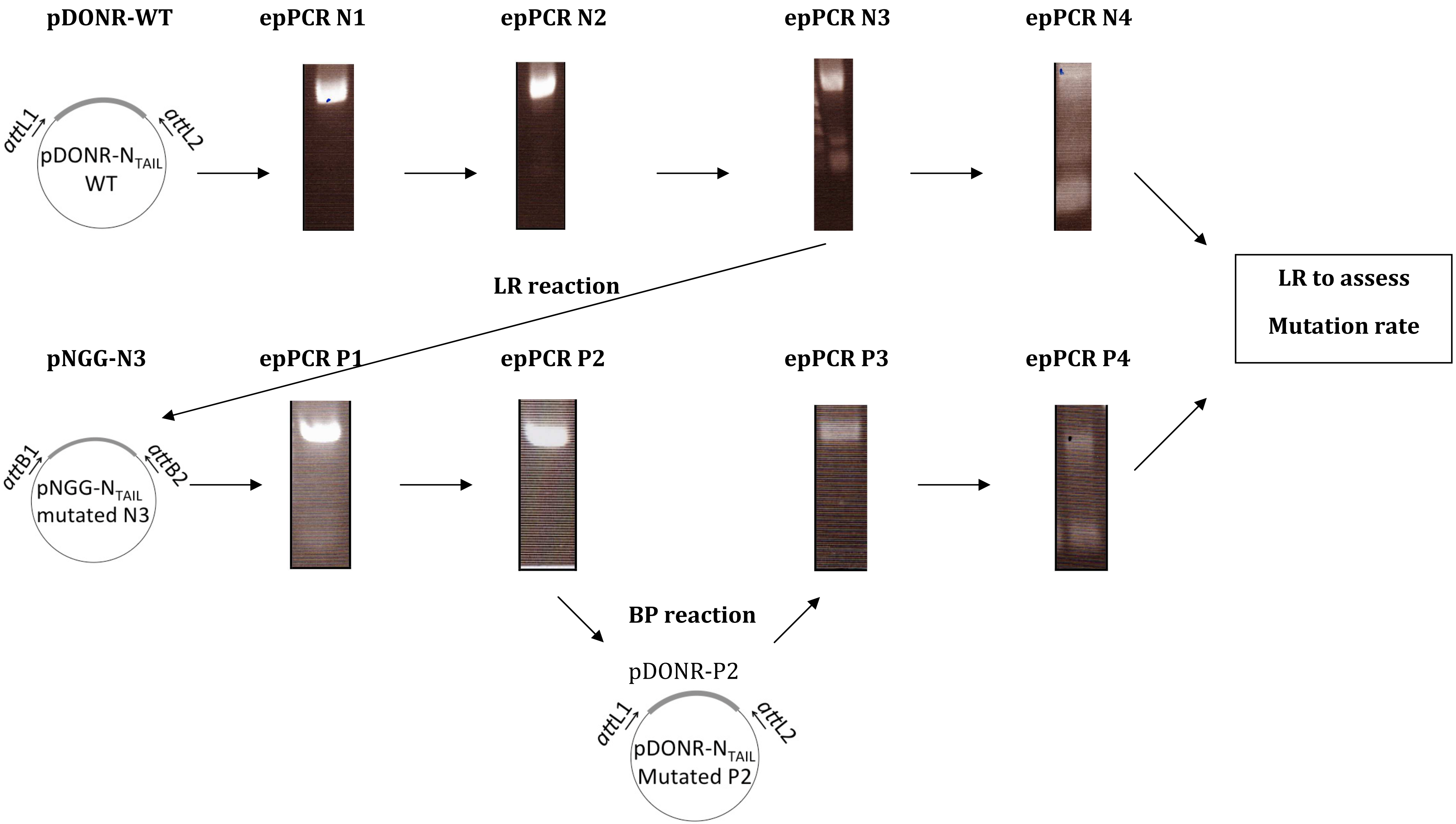
pDONR-WT
epPCR N1
epPCR N2
epPCR N3
epPCR N4
attL1
attL2
pDONR-N_{TAIL}
WT
LR reaction
LR to assess
Mutation rate
pNGG-N3
epPCR P1
epPCR P2
epPCR P3
epPCR P4
attB1
attB2
pNGG-N_{TAIL}
mutated N3
BP reaction
pDONR-P2
attL1
attL2
pDONR-N_{TAIL}
Mutated P2

Supplementary Figure S2. **Attempts to increase mutation rate above 1%**. A maximum average mutation rate of 1% base pairs was obtained although more than four iterative PCRs (in which 1 µl of the preceding PCR was used as template in the next one) had been performed to try to increase this rate, as suggested by Stratagene. As can be seen in this Figure, these successive PCR resulted in a smear in agarose gel electrophoresis. The main band started to smear with epPCR N3, and was totally replaced by incomplete products with epPCR N4. These incomplete DNA fragments proved to be a poor substrate for the LR reaction (*i.e.*, few clones on AKCplate). We reasoned that inserting a cloning step after epPCR N3 could "clean up" the smear by selecting the sole fragments that could be cloned, (*i.e.*, full length with correct ends). The product of epPCR N3 was thus cloned in pNGG by LR reaction. TAM1 cells were transformed with the LR reaction mix and then plated on Aplates. The pNGG-$_{NTAIL}$N3 plasmid mixture obtained by miniprep from the clones scraped off the plate was used as template in two additional epPCRs using the *att*B1 and *att*B2 primers. As can be seen, this PCR product no longer resolved as a smear, but as a definite band at the expected size (epPCR P1). The product of epPCR P2 was cloned into pDONR by a BP reaction. Two additional epPCRs were carried out using the resulting pENTR library (pDONR-N_{TAIL} Mutated P2) as template and the *att*L1 and *att*L2 primers. Following cloning of the mutated N_{TAIL} sequence in pNGG by LR, we assessed the mutation rate by sequencing 5 randomly chosen clones. Unfortunately, and quite surprisingly, we found that this procedure failed to yield an increased mutation rate. In conclusion: inserting a cloning step did solve the smear issue, but did not result in an increased mutation rate.
