# Supplementary material for: One-step generation of error-prone PCR libraries using Gateway® technology
Source: Microb Cell Fact. 2012 Jan 30;11:14. doi: 10.1186/1475-2859-11-14 (PMC3349575; doi:10.1186/1475-2859-11-14)
Supplement: Additional file 3 — Text S1. Nucleotide sequence of the coding sequences (S, N, 1-4) used in the experiments reported in Figure 4A and 4B. [file 1475-2859-11-14-S3.PDF]

>N

```
  1   K  K  A  G  S  T  T  E  D  K  I  S  R  A  V  G  P  R  Q  A
  1  AAAAAAGCAGGCTCGACTACTGAGGACAAGATCAGTAGAGCGGTTGGACCCAGACAAGCC

 21   Q  V  S  F  L  H  G  D  Q  S  E  N  E  L  P  R  L  G  G  K
 61  CAAGTATCATTTCTACACGGTGATCAAAGTGAGAATGAGCTACCGAGATTGGGGGGCAAG

 41   E  D  R  R  V  K  Q  S  R  G  E  A  R  E  S  Y  R  E  T  G
121  GAAGATAGGAGGGTCAAACAGAGTCGAGGAGAAGCCAGGGAGAGCTACAGAGAAACCGGG

 61   P  S  R  A  S  D  A  R  A  A  H  L  P  T  G  T  P  L  D  I
181  CCCAGCAGAGCAAGTGATGCGAGAGCTGCCCATCTTCCAACCGGCACACCCCTAGACATT

 81   D  T  A  S  E  S  S  Q  D  P  Q  D  S  R  R  S  A  D  A  L
241  GACACTGCATCGGAGTCCAGCCAAGATCCGCAGGACAGTCGAAGGTCAGCTGACGCCCTG

101   L  R  L  Q  A  M  A  G  I  S  E  E  Q  G  S  D  T  D  T  P
301  CTTAGGCTGCAAGCCATGGCAGGAATCTCGGAAGAACAAGGCTCAGACACGGACACCCCT

121   I  V  Y  N  D  R  N  L  L  D  -  -
361  ATAGTGTACAATGACAGAAATCTTCTAGACTAATAA
```

>S

```
  1   K  K  A  G  S  -  -
  1  AAAAAAGCAGGCTCTTAATAA
```

>1

```
  1   K  K  A  G  S  T  T  E  D  K  I  S  R  A  V  G  P  R  Q  A
  1  AAAAAAGCGGGCTCTACTACTGAGGACAAGATCAGTAGAGCGGTTGGACCCAGACAAGCC

 21   Q  V  S  F  L  H  G  D  Q  S  E  D  E  L  P  R  L  E  G  K
 61  CAAGTATCATTTCTACACGGTGATCAAAGTGAGGATGAGCTACCGAGATTGGAGGGCAAG

 41   E  D  R  R  V  K  Q  S  R  G  E  A  R  E  S  Y  R  E  T  G
121  GAAGATAGGAGGGTCAAACAGAGTCGAGGAGAAGCCAGGGAGAGCTACAGAGAAACCGGG

 61   P  S  R  A  S  D  A  R  A  A  H  L  P  T  G  I  P  L  D  I
181  CCCAGCAGAGCAAGTGATGCGAGAGCTGCCCATCTTCCAACCGGCATACCCCTAGATATT

 81   D  T  A  S  E  S  S  Q  D  P  Q  D  S  R  R  S  A  D  A  L
241  GACACTGCATCGGAGTCCAGCCAAGATCCGCAGGACAGTCGAAGGTCAGCTGACGCCCTG

101   L  R  L  Q  A  M  A  G  I  S  E  E  Q  G  S  D  T  D  T  P
301  CTTAGGCTGCAAGCCATGGCAGGAATCTCGGAAGAACAAGGCTCAGACACGGACACCCCT

121   I  V  Y  N  D  R  N  L  L  D  -  -
361  ATAGTGTACAATGACAGAAATCTTCTAGACTAATAA
```

>2

```
  1   K  K  A  G  S  T  T  E  D  K  I  S  R  A  V  G  P  R  Q  A
```

1 AAAAAAGCTGGCTCTACTACTGAGGACAAGATCAGTAGAGCGGTTGGACCCAGACAAGCC

21 Q V S F L H G D Q S E S E L P R L G G K
61 CAAGTATCATTTCTACACGGTGATCAAAGTGAGAGTGAGCTACCGAGATTGGGGGGCAAG

41 E D R R V K Q S R G E A R E S Y R E T G
121 GAAGATAGGAGGGTCAAACAGAGTCGAGGAGAAGCCAGGGAGAGCTACAGAGAAACCGGG

61 P S R A S D A R A A H L P T G T P L D I
181 CCCAGCAGAGCAAGTGATGCGAGAGCTGCCCATCTTCCAACCGGCACACCCCTAGACATT

81 D T A S E S S Q D P Q D S R R S A D A L
241 GACACTGCATCGGAGTCCAGCCAAGATCCGCAGGACAGTCGAAGGTCAGCTGACGCCCTG

101 L R L Q A M A G I S E E Q G S D T D T P
301 CTTAGGCTGCAAGCCATGGCAGGAATCTCGGAAGAACAAGGCTCAGACACGGACACCCCT

121 I V Y N D R N L L D - -
361 ATAGTGTACAATGACAGAAATCTTCTAGACTAATAA

>3
1 K K A G S T T E D K I S R A V G P R Q A
1 AAAAAAGCAGGCTCTACTACTGAGGACAAGATCAGTAGAGCGGTTGGACCCAGACAAGCC

21 Q V S F L H G D Q S E N E L P R L G G K
61 CAAGTATCATTTCTACACGGTGATCAAAGTGAGAATGAGCTACCGAGATTGGGGGGCAAG

41 E D R R V K Q S R G E A R E S Y R E T G
121 GAAGATAGGAGGGTCAAACAGAGTAGAGGAGAAGCCAGGGAGAGCTACAGAGAAACCGGG

61 P S R A S D A R A A H L P T G T P L D I
181 CCCAGCAGAGCAAGTGATGCGAGAGCTGCCCATCTTCCAACCGGCACACCCCTAGACATT

81 Y T A S E S S Q D P Q D S R R S A D A L
241 TACACTGCATCGGAGTCCAGCCAAGATCCGCAGGACAGTCGAAGGTCAGCTGACGCCCTG

101 L R L Q A M A G I S K E Q G S D T D T P
301 CTTAGGCTGCAAGCCATGGCAGGAATCTCGAAAGAACAAGGCTCAGACACGGACACCCCT

121 I V Y N D R N L L D E -
361 ATAGTGTACAATGACAGAAATCTTCTAGACGAATAA

>4
1 K K A G S T T E D K I S R A V G P R Q A
1 AAAAAAGCAGGCTCTACTACTGAGGACAAGATCAGTAGAGCGGTTGGACCCAGACAAGCC

21 Q V S F L H G D Q S E N E L P R L G G K
61 CAAGTATCATTTCTACACGGTGATCAAAGTGAGAATGAGCTACCGAGATTGGGGGGCAAG

41 E D R R V I Q S R G E A R E S Y R E T G
121 GAAGATAGGAGGGTCATACAGAGTCGAGGAGAAGCCAGGGAGAGCTACAGAGAAACCGGG

```
 61   P  N  R  V  S  D  A  R  A  A  H  L  P  T  G  T  P  L  D  I
181  CCCAACAGAGTAAGTGATGCGAGAGCTGCCCATCTTCCAACCGGCACACCCCTAGACATT

 81   D  T  A  S  E  S  S  Q  D  P  Q  D  S  -
241  GACACTGCATCGGAGTCCAGCCAAGATCCGCAGGACAGTTGAAGGTCAGCTGTCGCCCTG

301  CTTAGGCTGCAAGCCATGGCAGGAATCTCGGAAGAACAAGGCTCAGACACGGACACCCCT

361  ATAGTGTACAATGACAGAAATCTTCTAGACTAATAAA
```

Supplementary text S1. Nucleotide sequence and translation of the coding sequences (from top to bottom: S, N, 1 - 4) used in the experiments reported in Figure 4A and B.
