# Supplementary material for: One-step generation of error-prone PCR libraries using Gateway® technology
Source: Microb Cell Fact. 2012 Jan 30;11:14. doi: 10.1186/1475-2859-11-14 (PMC3349575; doi:10.1186/1475-2859-11-14)
Supplement: Additional file 4 — Table S1. This table compares the experimental conditions of experiment I, II and III reported in Figure 4C. [file 1475-2859-11-14-S4.PDF]

| Experiment | | | I | II and III |
|---|---|---|---|---|
| PCR1 | Template | | 1µl | 0.25µl |
| | Volume | | 50µl | 2 x 50µl |
| | 10µM each primer | | 1µl | 10µl |
| | Cycles | | 10 | 30 |
| | DpnI | Volume | 1µl | 2 x 2µl |
| | | Incubation at 37°C | 2 hours | O/N |
| | Final volume | | 50µl | |
| PCR2 | Template | | 3µl | 8µl |
| | Megaprimers | | 25µl | 20µl |
| | Cycles | | 20 | |
| | DpnI | | 1µl O/N | |
| Colonies | | | 10 | >100 |

**Supplementary Table S1**. This table compares the experimental conditions of experiments I, II and III reported in Figure 4C. "PCR1" and "PCR2" refer to Figure 1 (stage 2, right flowchart), and to Figure 2. In PCR1, "Template" is the volume of mutated pNGG-$N_{TAIL}$ (plasmid mini-preparation at a concentration of 62 ng/µl). "Volume" indicates the total volume of PCR in that experiment. "10 µM each primer" is the stock concentration of each primer (*att*B1 and *att*B2) used in PCR1. "Cycles" is the number of PCR cycles. "DpnI": the volume of a 20U/µl DpnI solution and the incubation time at 37°C are indicated. "O/N" stands for "overnight incubation". In PCR2, "Template" is the volume of either full length $N_{TAIL}$ borne by pDEST17O/I (pDEST17O/I-$N_{TAIL}$ (Table 5) plasmid mini-preparation at a concentration of 30 ng/µl) (experiment II) or internally deleted (227 bp deletion) $N_{TAIL}$ borne by pDEST17O/I (pDEST17O/I-id$N_{TAIL}$ (Table 5) plasmid mini-preparation at a concentration of 103 ng/µl) (experiments I and III). "Megaprimers" indicates the volume of megaprimers used in PCR2, *i.e.* the volume uptaken from PCR1 after DpnI treatment and DNA purification. "Colonies" is the number of colonies counted on ampicillin plate after transformation of 50 µl of TAM1 cells with 5 µl of PCR2 mixture after DpnI treatment.
