# Supplementary material for: One-step generation of error-prone PCR libraries using Gateway® technology
Source: Microb Cell Fact. 2012 Jan 30;11:14. doi: 10.1186/1475-2859-11-14 (PMC3349575; doi:10.1186/1475-2859-11-14)
Supplement: Additional file 5 — Figure S3. Optimization of co-expression conditions. [file 1475-2859-11-14-S5.PDF]

	Temperature (°C)	Medium	Strain	IPTG (µM)	Arabinose (%)
A	37	TB	BL21 pLysS (B)	500	2
	25	2YT	Rosetta pLysS (R)	100	0.2
	17	LB	T7 pRos (T)	10	0.02
			C41 pRos (C)		

	Temperature (°C)	Medium	Strain	IPTG (µM)	Arabinose (%)	Condition
B	17	LB	C	10	0.2	1
	25	LB	T	500	0.02	2
	17	2Y	B	10	0.02	3
	17	2Y	B	100	2	4
	37	2Y	T	10	2	5
	25	2Y	R	500	0.2	6
	37	TB	B	10	0.2	7
	37	LB	T	100	0.02	8
	17	TB	T	100	0.2	9
	37	LB	B	500	0.02	10
	25	LB	B	500	2	11
	25	TB	C	100	2	12
	37	2Y	C	500	0.2	13
	37	TB	R	500	2	14
	25	TB	B	100	0.2	15
	25	2Y	C	100	0.02	16
	25	2Y	T	10	0.2	17
	25	TB	R	10	0.02	18
	17	TB	C	500	0.02	19
	37	2Y	R	100	0.02	20
	17	TB	T	500	2	21
	37	LB	C	10	2	22
	17	LB	R	100	0.2	23
	17	LB	R	10	2	24

	Temperature (°C)	Medium	Strain	IPTG (µM)	Arabinose (%)	Condition
C	**17**	**TB**	**T**	**500**	**2**	**Best**

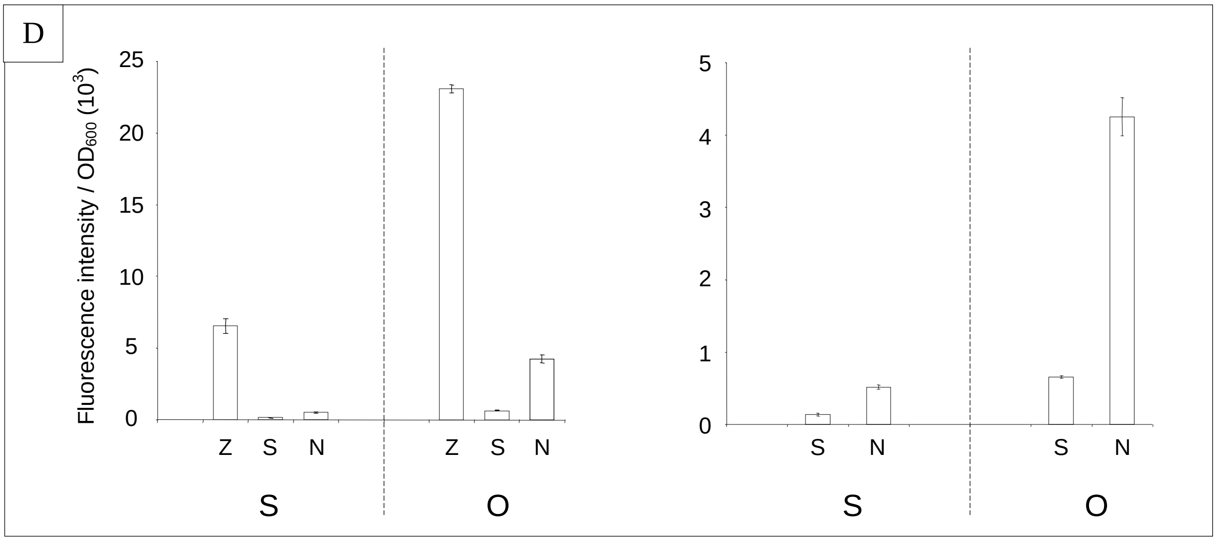

Supplementary Figure S3. Optimization of co-expression conditions. T7pRos cells were co-transformed as described in Methods (*Protein expression and purification*). After induction, the cells were recovered by centrifugation, and their fluorescence measured as described in Methods (*Library screening*). The best co-expression conditions were determined by using a fractional factorial approach modified from Benoit *et al*. (2007). In practice, two variables were added, namely expression inducers IPTG and arabinose. The complete list of the variables tested is reported in table A. The full factorial was made of 324 combinations. Using the SAmBA online freeware (http://www.igs.cnrs-mrs.fr/samba/), these were reduced to 24 (table B). The fluorescence data provided by each of these 24 combinations were recorded (not illustrated), and the best combination (table C) was found by performing a trend analysis as described in Benoit *et al*. (2007). In D, we have compared the fluorescence results provided by the standard conditions used by Magliery *et al.* (2005) (S) to those optimized by the fractional factorial approach (O). Three control constructs were used: the anti-parallel leucine zippers (Z), *wt*N_{TAIL} (N) and Stop-N_{TAIL} (S). In the graph on the right, Z was omitted to make the comparison of N and S more readable. Abbreviations. LB, Luria-Bertani. TB, Terrific broth.

References

Benoit, I., Coutard, B., Oubelaid, R., Asther, M. and Bignon, C. (2007) Expression in Escherichia coli, refolding and crystallization of Aspergillus niger feruloyl esterase A using a serial factorial approach. *Protein Expr Purif*, **55**, 166-174.

Magliery, T.J., Wilson, C.G., Pan, W., Mishler, D., Ghosh, I., Hamilton, A.D. and Regan, L. (2005) Detecting protein-protein interactions with a green fluorescent protein fragment reassembly trap: scope and mechanism. *J Am Chem Soc*, **127**, 146-157.
